# Situations Leading to Reduced Effectiveness of Current Hand Hygiene against Infectious Mucus from Influenza Virus-Infected Patients

**DOI:** 10.1128/mSphere.00474-19

**Published:** 2019-09-18

**Authors:** Ryohei Hirose, Takaaki Nakaya, Yuji Naito, Tomo Daidoji, Risa Bandou, Ken Inoue, Osamu Dohi, Naohisa Yoshida, Hideyuki Konishi, Yoshito Itoh

**Affiliations:** a Department of Molecular Gastroenterology and Hepatology, Graduate School of Medical Science, Kyoto Prefectural University of Medicine, Kyoto, Japan; b Department of Infectious Diseases, Graduate School of Medical Science, Kyoto Prefectural University of Medicine, Kyoto, Japan; c Department of Forensics Medicine, Graduate School of Medical Science, Kyoto Prefectural University of Medicine, Kyoto, Japan; National Institute of Allergy and Infectious Diseases

**Keywords:** hand hygiene, antiseptic hand rubbing, antiseptic hand washing, ethanol-based disinfectant, viscosity, mucus, sputum, influenza A virus, diffusion, fluid simulation, disinfectants, influenza

## Abstract

Both antiseptic hand rubbing (AHR) using ethanol-based disinfectants (EBDs) and antiseptic hand washing (AHW) are important means of infection control to prevent seasonal influenza A virus (IAV) outbreaks. However, previous reports suggest a reduced efficacy of ethanol disinfection against pathogens in mucus. We aimed to elucidate the situations and mechanisms underlying the reduced efficacy of EBDs against IAV in infectious mucus. We evaluated IAV inactivation and ethanol concentration change using IAV-infected patients’ mucus (sputum). Additionally, AHR and AHW effectiveness against infectious mucus adhering to the hands and fingers was evaluated in 10 volunteers. Our clinical study showed that EBD effectiveness against IAV in mucus was extremely reduced compared to IAV in saline. IAV in mucus remained active despite 120 s of AHR; however, IAV in saline was completely inactivated within 30 s. Due to the low rate of diffusion/convection because of the physical properties of mucus as a hydrogel, the time required for the ethanol concentration to reach an IAV inactivation level and thus for EBDs to completely inactivate IAV was approximately eight times longer in mucus than in saline. On the other hand, AHR inactivated IAV in mucus within 30 s when the mucus dried completely because the hydrogel characteristics were lost. Additionally, AHW rapidly inactivated IAV. Until infectious mucus has completely dried, infectious IAV can remain on the hands and fingers, even after appropriate AHR using EBD, thereby increasing the risk of IAV transmission. We clarified the ineffectiveness of EBD use against IAV in infectious mucus.

**IMPORTANCE** Antiseptic hand rubbing (AHR) and antiseptic hand washing (AHW) are important to prevent the spread of influenza A virus (IAV). This study elucidated the situations/mechanisms underlying the reduced efficacy of AHR against infectious mucus derived from IAV-infected individuals and indicated the weaknesses of the current hand hygiene regimens. Due to the low rate of diffusion/convection because of the physical properties of mucus as a hydrogel, the efficacy of AHR using ethanol-based disinfectant against mucus is greatly reduced until infectious mucus adhering to the hands/fingers has completely dried. If there is insufficient time before treating the next patient (i.e., if the infectious mucus is not completely dry), medical staff should be aware that effectiveness of AHR is reduced. Since AHW is effective against both dry and nondry infectious mucus, AHW should be adopted to compensate for these weaknesses of AHR.

## Abstract (precis)

Antiseptic hand rubbing (AHR) and antiseptic hand washing (AHW) are important to prevent the spread of influenza A virus (IAV). This study elucidated the situations/mechanisms underlying the reduced efficacy of AHR against infectious mucus derived from IAV-infected individuals and indicated the weaknesses of the current hand hygiene regimens. Due to the low rate of diffusion/convection because of the physical properties of mucus as a hydrogel, the efficacy of AHR using ethanol-based disinfectant against mucus is greatly reduced until infectious mucus adhering to the hands/fingers has completely dried. If there is insufficient time before treating the next patient (i.e., if the infectious mucus is not completely dry), medical staff should be aware that effectiveness of AHR is reduced. Since AHW is effective against both dry and nondry infectious mucus, AHW should be adopted to compensate for these weaknesses of AHR.

## INTRODUCTION

Prevention of influenza A virus (IAV) transmission during annual outbreaks of seasonal influenza is a major issue (1–3). Although droplet infection is most frequent, contact infection is also an important infection route (1, 4–6). Hand hygiene is a commonly used method for prevention of contact infection and is recommended by the Centers for Disease Control and Prevention (CDC) and the World Health Organization (WHO) (7–10). There are two recognized techniques for performing hand hygiene: antiseptic hand rubbing (AHR) with alcohol-based disinfectants, such as 80% (wt/wt) ethanol-based disinfectants (EBDs), and antiseptic hand washing (AHW) (11).

EBDs are promoted based on the premise that they inactivate IAV in infectious mucus. While the effect of EBDs on pathogens in mucus such as sputum is reportedly satisfactory in actual use (12, 13), studies show that organic matter such as mucosal proteins may decrease this effectiveness (14–16). Since organic matter contained in hand dirt or body fluids may reduce EBD effectiveness, methods and standards for evaluating disinfectant efficacy by adding organic matter as a load material have been established by the American Society for Testing and Materials (ASTM) and the European Committee for Standardization (CEN) (17–20). EBDs comply with these standards and are used in medical institutions around the world.

We previously reported that EBD effectiveness against IAV in mucus may be reduced due to physical factors (e.g., viscoelasticity) and not chemical factors (e.g., organic matter) (21, 22). Disinfectant effectiveness against bacteria in biofilms decreases due to the physical factors of biofilms (23–25). Because mass transfer is limited in biofilms (26, 27), a disinfectant solution takes a long time to reach the center of a biofilm. Therefore, bacteria in biofilms are protected from disinfectants and survive longer (26, 28–30). Human mucus, including sputum, is a viscoelastic substance that shares physical properties with biofilms (31). Therefore, mucus may also protect IAV from disinfectants due to its physical properties.

Disinfectant effectiveness against IAV in mucus has not been evaluated based on the physical properties of mucus, and the mechanisms mediating IAV survival in mucus are yet unknown. Elucidation of these mechanisms will help clarify the limitations of current AHR regimens in disinfecting infectious mucus. Assessment of disinfectants in the presence of mucus may help the development of effective disinfectants. With identification of conditions leading to attenuation of the effectiveness of disinfectants, more effective AHR methods can be proposed.

In this study, we aimed to demonstrate the limited effectiveness of EBD against IAV in mucus (sputum). We further sought to elucidate the mechanisms responsible for this limitation and the situations where this limitation occurs.

## RESULTS

### Physical properties and complete drying time of mucus.

**FIG 1:**
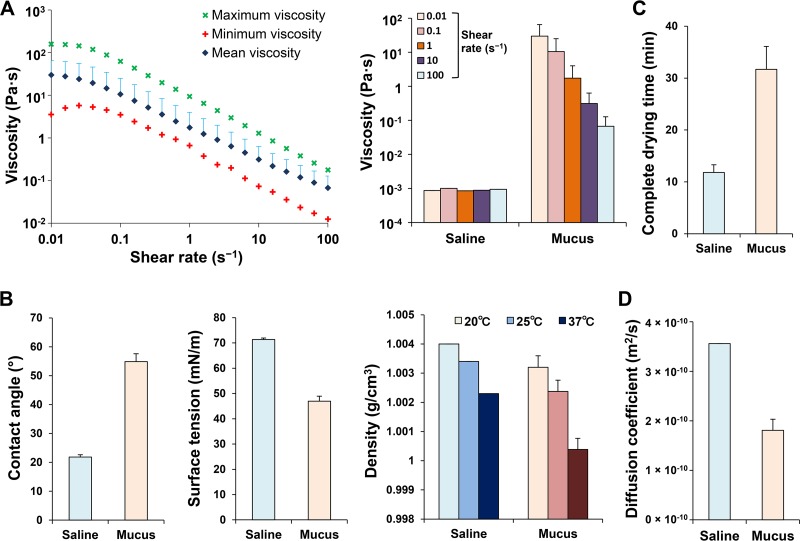
Analysis of the physical properties of mucus from 19 influenza A virus-infected patients. (A) Rheological analysis: mean, maximum, and minimum viscosities of mucus (sputum) samples at different shear rates (left panel). Shown is a comparison of mucus and saline viscosities at different shear rates (right panel). (B) Average parameters measured for saline and mucus samples: contact angles, surface tension, and densities at different temperatures. (C) Time required for saline and mucus to completely dry (complete drying time). (D) Ethanol diffusion coefficients of saline and mucus exposed to an 80% ethanol concentration.

We performed a rheological analysis of the fluid flow properties of saline and the mucus samples (see Table S1 in the supplemental material). All mucus samples showed characteristics of a pseudoplastic fluid (a fluid whose viscosity decreases as the shear-rate increases). The viscosity of the mucus was significantly higher than that of saline (*P* < 0.001) at all shear rates (0.01 to 100 s^−1^ [Fig. 1A]).

10.1128/mSphere.00474-19.7 TABLE S1 Viscosity of mucus (19 mucus samples). Download Table S1, DOCX file, 0.02 MB. Copyright © 2019 Hirose et al. 2019 Hirose et al. This content is distributed under the terms of the Creative Commons Attribution 4.0 International license.

We also measured other physical properties (Fig. 1B; see Fig. S1 in the supplemental material). The mean contact angle (the angle between the liquid and contact surfaces) of the mucus samples was higher than that of saline (54.9 ± 2.7 versus 21.8 ± 0.8°; *P* < 0.001). Additionally, the mean surface tension of the mucus samples was lower than that of saline (46.9 ± 2.0 versus 71.4 ± 0.6 mN/m; *P* < 0.001), and the density of the mucus samples was lower than that of saline in the normal temperature range of 20 to 37°C.

10.1128/mSphere.00474-19.1 FIG S1 Analysis of the physical properties of mucus. (A) Contact angles of saline and mucus (19 sputum samples) on a glass surface were measured with a contact angle meter. (B) Surface tension of saline and mucus samples was measured with a surface tension meter. (C) Density of the saline and mucus samples was measured at 20, 25, and 37°C. (D) Time required for 5 μl of saline and mucus samples to completely dry and solidify on a glass plate (complete drying time). Download FIG S1, TIF file, 1.1 MB. Copyright © 2019 Hirose et al. 2019 Hirose et al. This content is distributed under the terms of the Creative Commons Attribution 4.0 International license.

Furthermore, the time required for 5 μl of saline or mucus samples to completely dry and solidify on a glass plate was measured. The mean complete drying time of the mucus samples was longer than that of saline (33.1 ± 2.7 versus 18.7 ± 0.8 min; *P* < 0.001 [Fig. 1C]).

### Ethanol diffusion in mucus.

Coefficients were determined for ethanol concentrations of 10 to 80%, and a fitting curve was prepared from the values (see Fig. S2 in the supplemental material). In the presence of 80% ethanol, the mean diffusion coefficient in mucus was 1.8 × 10^−10^ ± 6.9 × 10^−11^ m^2^/s, and the coefficient in saline was 3.6 × 10^−10^ m^2^/s (Fig. 1D).

10.1128/mSphere.00474-19.2 FIG S2 Evaluation of the ethanol diffusion coefficient in mucus. Three representative mucus samples were selected (no. 1, 10, and 19). Ethanol diffusion coefficients in mucus and saline samples were measured by pulsed-field gradient nuclear magnetic resonance (PFG-NMR). Fitting curves of ethanol diffusion coefficients were prepared from the measurements (top panel). Diffusion coefficients for each ethanol concentration calculated from the fitting curve are shown (bottom table). Download FIG S2, TIF file, 1.0 MB. Copyright © 2019 Hirose et al. 2019 Hirose et al. This content is distributed under the terms of the Creative Commons Attribution 4.0 International license.

In a simulation of changes in ethanol concentration that was conducted in Open-source Field Operation And Manipulation (OpenFOAM) software, considering only diffusion phenomena (and using the diffusion coefficients shown in Fig. S2), the ethanol concentration increase rate (ECIR) was indeed lower in mucus than in saline (see Fig. S3 in the supplemental material).

10.1128/mSphere.00474-19.3 FIG S3 Simulation of changes in ethanol concentration considering only diffusion phenomena using OpenFOAM software. (A) The ethanol interdiffusion coefficient of saline (left panel) and the average ethanol interdiffusion coefficients of three mucus samples (right panel) were used for the simulation. (B) Initial placement of air, 80% ethanol, and saline/mucus in this diffusion evaluation model. (C) Overview of the changes in ethanol concentration by simulation under saline conditions. (D) Changes in ethanol concentration near the boundary between saline and 80% ethanol were simulated (ethanol concentration immediately after the start of the simulation [left panel] and 60 s after simulation initiation [right panel]). The region indicated by a light green arrow in these graphs is the same region as that indicated by a light green arrow in panels B and C. (E) Changes in ethanol concentration near the boundary between the mucus and 80% ethanol samples were simulated (ethanol concentration immediately after the start of simulation [left panel] and 60 s after simulation initiation [right panel]). Download FIG S3, TIF file, 2.4 MB. Copyright © 2019 Hirose et al. 2019 Hirose et al. This content is distributed under the terms of the Creative Commons Attribution 4.0 International license.

### Measurement of the changes in ethanol concentrations in mucus.

**FIG 2:**
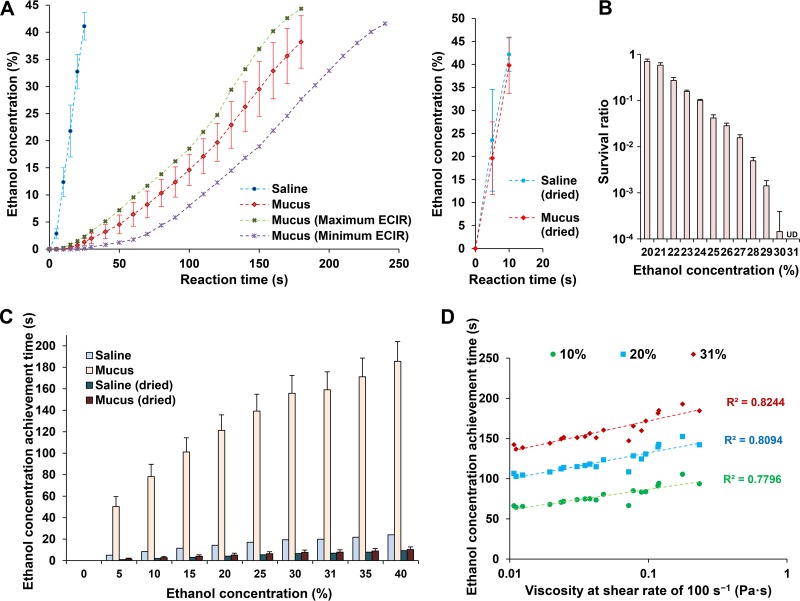
Changes in ethanol concentration over time. (A, left panel) Time course showing ethanol concentrations in the center region of saline and mucus samples exposed to ethanol-based disinfectant (EBD) (mean ± standard deviation [SD]). (A, right panel) Time course of ethanol concentrations in the center region of dried saline and dried mucus samples exposed to EBD. (B) Influenza A virus survival in saline with different ethanol concentrations. (C) Times at which saline and mucus exposed to EBD reached specific ethanol concentrations (mean ± SD; *n* = 3). (D) Scatter plot of mucus viscosity at shear rate of 100 (1/s) versus time for ethanol concentration in mucus to reach 10, 20, and 31% (Pearson’s correlation coefficient analysis).

With a saline sample, the ethanol concentration increased to >40% within 30 s, whereas the ethanol concentration in the mucus samples reached 40% by ∼180 s, demonstrating that the ECIR in mucus was lower than that in saline (Fig. 2A). However, in completely dried saline and dried mucus samples, the ethanol concentration increased to >40% in approximately 10 s, and the ECIRs were comparable between the two sample types (Fig. 2A). Furthermore, our data show that IAV used in this study was completely inactivated after exposure to ≥31% ethanol for 30 s (Fig. 2B). Therefore, the minimum ethanol concentration that can completely inactivate IAV was set to 31% in this study.

The mean times required for the ethanol concentration to reach 31% were 19.9 s for saline, 159.0 s for mucus, 6.9 s for dried saline, and 7.9 s for dried mucus (Fig. 2C). The time required for the ethanol concentration to reach 31% was approximately 8 times longer in mucus than in saline. Moreover, viscosity (100 s^−1^) correlated positively with the time required for the ethanol concentration to reach 31%, with a correlation coefficient of 0.908 (*P* = 0.001) (Fig. 2D).

### Initial fluid simulation of the changes in ethanol concentrations in mucus.

**FIG 3:**
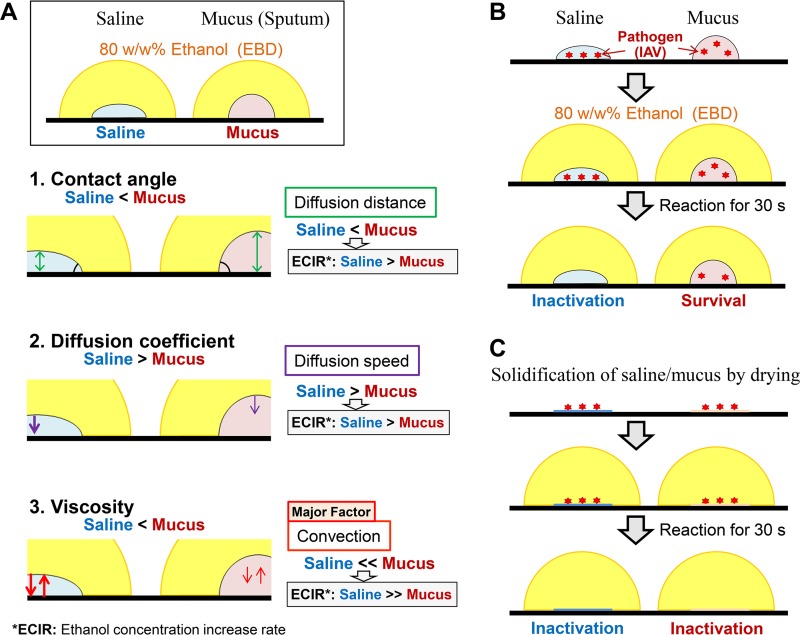
Models of the influence of the physical properties of saline and mucus on the ethanol concentration increase rate (ECIR). (A) Based on the physical properties (contact angle and viscosity) and ethanol diffusion coefficients of mucus and saline, it was predicted that ECIR would be considerably lower in mucus than in saline. (B) Due to the lower ECIR in mucus, pathogens in mucus survive longer than in saline after exposure to ethanol-based disinfectant (EBD). (C) When saline or mucus is completely dried and solidified, EBD acts quickly to inactivate the pathogen.

**FIG 4:**
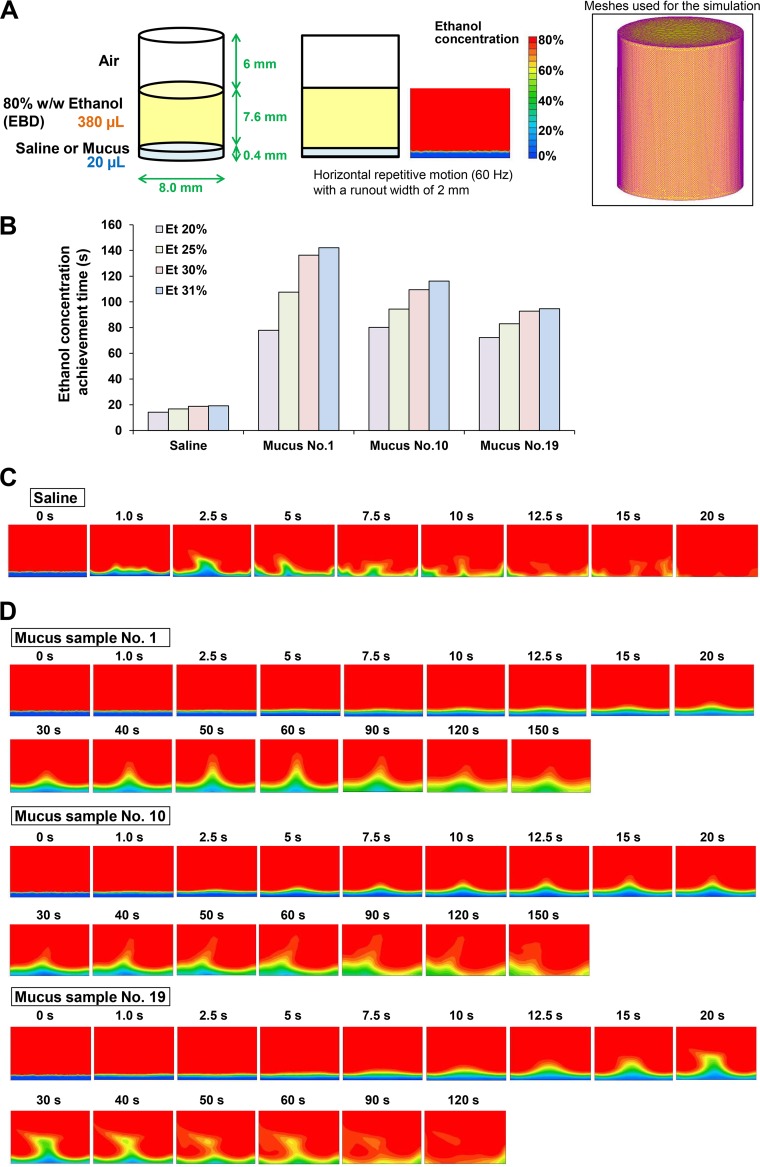
Initial fluid simulation of the changes in ethanol concentrations in mucus. (A) The initial simulation reproducing the mixing of 20 μl of saline/mucus and 380 μl of EBD in a well of a 96-well plate was conducted. Figure S6 shows the physical characteristic values used for this fluid analysis. (B) Times at which the lowest ethanol concentration in the whole region reached 20, 25, 30, and 31% were calculated by the initial fluid simulation. (C) Simulation of change in ethanol concentration in saline produced by adding EBD (80% ethanol). (D) Simulation of the change in ethanol concentration in mucus samples (sample no. 1, 10, and 19) produced by adding EBD.

The differences in contact angle, viscosity, and diffusion coefficient should each result in a lower ECIR for mucus than for saline (Fig. 3A). Fluid simulation was conducted using all of the measured physical property values and diffusion coefficients to demonstrate that the physical properties of mucus greatly affect the ECIR in mucus (Fig. 4).

In the saline mixture, the ethanol concentration in all regions increased to ≥31% in 19.1 s. In contrast, in the mucus mixture with sample no. 1, 10, and 19, the ethanol concentration in all regions increased to ≥31% in 142.1, 116.2, and 94.7 s, respectively. The time required for the ethanol concentration to reach 31% was approximately 5 to 8 times longer in mucus than in saline, which was relatively consistent with the results of ECIR analysis by ethanol concentration measurement (Fig. 2C and Fig. 4B).

### *In vitro* inactivation tests for evaluation of EBD efficacy.

**FIG 5:**
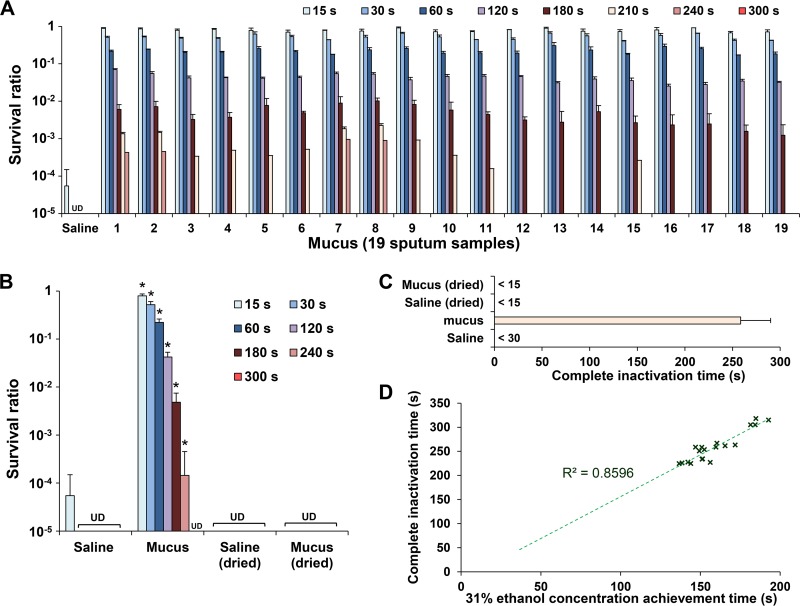
*In vitro* inactivation tests of ethanol-based disinfectant (EBDs) against influenza A virus (IAV). IAV-containing saline or mucus samples were exposed to EBD or PBS for 15 to 300 s. Survival ratios of IAV were determined from viral titers in EBD-treated versus PBS-treated samples. (A) Survival ratios of IAV in saline and mucus samples after exposure to EBDs. (B) Mean survival ratios of IAV in saline, mucus, dried saline, and dried mucus (expressed as mean ± SD; *n* = 3). UD, undetectable. *, *P* < 0.005. (C) Time taken for complete inactivation (survival ratio of <10^−5^) of IAV by EBD in saline, mucus, dried saline, and dried mucus. (D) Scatter plot of “time for ethanol concentration in mucus to reach 31%” versus “complete inactivation time for IAV in mucus” (Pearson’s correlation coefficient analysis).

In a control experiment, IAV in 5 μl saline was completely inactivated within 30 s of exposure to 95 μl EBD containing 80% (wt/wt) ethanol. In contrast, IAV in all 19 mucus samples survived exposure to EBD for more than 180 s (Fig. 5A). The IAV survival ratio (survival following incubation with EBD relative to survival following incubation with phosphate-buffered saline [PBS]) was significantly higher in mucus than in saline at all exposure times (*P* < 0.005 for all comparisons [Fig. 5B]). The time required for EBD to completely inactivate IAV (the complete inactivation time) in mucus was approximately eight times longer than it was in saline (258.5 ± 31.4 versus <30 s [Fig. 5C]). Moreover, the complete inactivation time correlated positively with the time required for the ethanol concentration to reach 31%, with a correlation coefficient of 0.927 (*P* = 0.001) (Fig. 5D). Interestingly, both IAV in dried saline and IAV in dried mucus were inactivated rapidly within 15 s (Fig. 5B and C). These results were in agreement with the results of ECIR analysis by ethanol concentration measurement in saline and mucus (Fig. 2C and Fig. 3).

### Clinical research on the efficacy evaluation of AHR or AHW.

The clinical analysis showed that although IAV in saline was completely inactivated by AHR using EBD within 30 s, IAV in all mucus samples remained active, even after AHR for 120 s, and was completely inactivated by AHR within 240 s. The log reduction of IAV in mucus was significantly lower than that in saline after AHR for 30, 60, and 120 s (i.e., the IAV survival ratio in mucus was significantly higher than that in saline). The efficacy of AHR was also examined after allowing the saline and mucus samples to dry completely after placing them on the fingers. IAV was inactivated rapidly by AHR within 30 s in both dried mucus samples and dried saline. Furthermore, IAV was inactivated rapidly by AHW within 30 s under all conditions (in both undried and dried mucus samples and undried and dried saline [Table 1]).

**TABLE 1:** Log reduction of IAV in mucus or saline caused by AHR or AHW

Sample	Disinfection time (s)	IAV log reduction by condition^*a*^				
		Nondrying			Drying	
		AHR	*P*	AHW	AHR	AHW
Saline	30	>5		>5	>5	>5
	60	>5		>5	>5	>5
	120	>5		>5	>5	>5
	240	>5		>5	>5	>5

Mucus						
All samples	30	0.84 ± 0.28	0.002	>5	>5	>5
	60	1.87 ± 0.33	0.002	>5	>5	>5
	120	2.95 ± 0.36	0.002	>5	>5	>5
	240	>5		>5	>5	>5
Each sample						
No. 1	30	0.70 ± 0.49	0.002	>5	>5	>5
	60	1.55 ± 0.55	0.002	>5	>5	>5
	120	3.00 ± 1.14	0.008	>5	>5	>5
	240	>5		>5	>5	>5
No. 10	30	0.66 ± 0.30	0.002	>5	>5	>5
	60	1.86 ± 0.82	0.002	>5	>5	>5
	120	2.57 ± 0.62	0.002	>5	>5	>5
	240	>5		>5	>5	>5
No. 19	30	1.17 ± 0.38	0.002	>5	>5	>5
	60	2.21 ± 0.67	0.002	>5	>5	>5
	120	3.29 ± 0.75	0.004	>5	>5	>5
	240	>5		>5	>5	>5

a IAV, influenza A virus; AHR, antiseptic hand rubbing; AHW, antiseptic hand washing. Statistics are presented as mean ± SD with the Wilcoxon signed-rank test. The log reduction values between saline and mucus conditions were compared.

### Additional fluid simulation of the changes in ethanol concentrations in mucus.

Finally, we tried to simulate the reaction conditions of the ethanol concentration measurement and the inactivation tests more faithfully.

**FIG 6:**
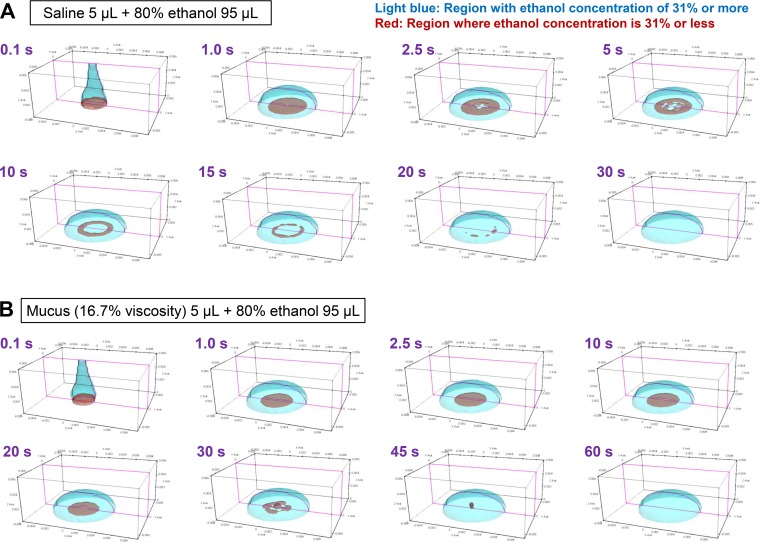
Additional fluid simulation of changes in the ethanol concentrations in mucus. (A) Addition of 95 μl of 80% ethanol to 5 μl saline was modeled in OpenFOAM, demonstrating the disappearance over time of the region with <31% ethanol (shown in red). See also Movie S1. (B) Addition of 95 μl of 80% ethanol to 5 μl mucus was modeled. The simulation was performed under conditions of reduced viscosity (16.7% of the actual measurement value) because of the long time required for disappearance of the concentration region of <31%. See also Movie S2.

In the simulated mixing of saline and EBD, the ethanol concentration in all regions increased to ≥31% within 30 s (Fig. 6A; see Movie S1 in the supplemental material). Simulations for mucus mixtures were performed with mucus viscosity reduced to 16.7 or 12.5% of the measured value (see Materials and Methods for an explanation of the reason). In this simulation, the central region of the mucus remained at <31% ethanol for >30 s indicating a low ECIR, which corresponded with the experimental results (Fig. 6B; see Movie S2 in the supplemental material). All low-ethanol regions disappeared by ∼60 s. Running of the simulation with a mucus viscosity at 16.7 or 12.5% of the measured value demonstrated that the ECIR was lower at the higher viscosity (see Fig. S4A in the supplemental material), suggesting that viscosity was a determining factor for ECIR in mucus and that this affects the level of mixing between EBD and mucus by convection.

10.1128/mSphere.00474-19.4 FIG S4 A supplemental study to evaluate the validity of the fluid simulation. (A) Times at which the lowest ethanol concentration in the whole region reached 20, 25, 30, and 31% in the additional fluid simulation models of saline and mucus (at 12.5 or 16.7% of the measured viscosity). (B) Changes in ethanol concentrations measured using a modified digital ethanol densitometer; the time course shows ethanol concentrations in the region with the lowest ethanol concentration of reference samples at 12.5 and 16.7% of the standard mucus (sample no. 10) viscosity. (C) Actual measurement of times at which the lowest ethanol concentration in the entire region reached 20, 25, 30, and 31% in saline, the reference samples (with 12.5 and 16.7% viscosity), and standard mucus. (D) Times at which the lowest ethanol concentration in the whole region reached 20, 25, 30, and 31% in the initial fluid simulation models of saline and mucus (at 12.5 or 16.7% of the measured viscosity). Download FIG S4, TIF file, 1.0 MB. Copyright © 2019 Hirose et al. 2019 Hirose et al. This content is distributed under the terms of the Creative Commons Attribution 4.0 International license.

10.1128/mSphere.00474-19.8 MOVIE S1 Addition of 95 μl of 80% ethanol to 5 μl saline was modeled in OpenFOAM, demonstrating the disappearance over time of the region with <31% ethanol (shown in red). Download Movie S1, MPG file, 5.6 MB. Copyright © 2019 Hirose et al. 2019 Hirose et al. This content is distributed under the terms of the Creative Commons Attribution 4.0 International license.

10.1128/mSphere.00474-19.9 MOVIE S2 Addition of 95 μl of 80% ethanol to 5 μl mucus was modeled. The simulation was performed under conditions of reduced viscosity (16.7% of the actual measurement value) because of the long time required for disappearance of the concentration region of <31%. Download Movie S2, MPG file, 8.4 MB. Copyright © 2019 Hirose et al. 2019 Hirose et al. This content is distributed under the terms of the Creative Commons Attribution 4.0 International license.

As a supplemental study to evaluate the validity of this fluid simulation, low-viscosity mucus (12.5 and 16.7% of the measured value) with physical properties almost similar to those applied to this simulation was prepared by dilution of a mucus sample with water, and the time course of the change in ethanol concentration following addition of the EBD was measured. The ethanol concentration at low mucus viscosities of 12.5 and 16.7% increased to 31% in 45.9 and 51.8 s, respectively (Fig. S4B), which was generally compatible with the results of this fluid simulation (Fig. S4A and C). Moreover, the result of the initial fluid simulation, which was conducted under the same low-viscosity conditions as this additional simulation, was generally compatible with the results of this fluid simulation (Fig. S4A and D).

## DISCUSSION

It is widely known that the effects of disinfectants such as EBDs are attenuated by organic matter contained in body fluids: thus, evaluation of EBD effectiveness after adding organic matter is included in the current disinfectant effectiveness evaluation standards (15, 17–20). Although several studies have evaluated and discussed the effectiveness of disinfectants on pathogens in infectious mucus such as sputum, major factors other than organic matter, which may reduce the effectiveness of disinfectants, have not been identified (12–14, 16). In this study, we focused on IAV, which is the most common and important pathogen among those causing respiratory tract infections and those spread by infectious mucus such as sputum. Building further on our previous research (22), we elucidated the situations and mechanisms in which the effectiveness of EBD against IAV in infectious mucus is extremely weakened, and we clarified the weak points of current AHR regimens and contact infection prevention strategies.

Based on the fluid simulations using measured physical properties and diffusion coefficients, the ECIR in mucus was expected to be much slower than the ECIR in saline (Fig. 3A). In fact, measurement of the ethanol concentration in saline and mucus revealed that the time required for the ethanol concentration to reach 31% (i.e., the minimum concentration at which IAV is completely inactivated) throughout the mucus was approximately eight times longer than that in saline; this positively correlated with IAV inactivation time. Taken together, these findings revealed that ECIR in infectious mucus was considerably low; this was because of decreased diffusion and convection phenomena due to the unique physical properties (particularly the high viscosity) of infectious mucus, which caused prolongation of the time required for EBD to exert its disinfecting effects on IAV in infectious mucus.

In this clinical study, infectious mucus prepared from sputum samples collected from IAV-infected patients was applied to human fingers, and *in situ* inactivation tests were performed. We aimed to reproduce the situation in which infectious mucus discharged from IAV-infected patients adheres to the fingers of medical staff. There was close agreement between inactivation test results obtained from *in situ* and *in vitro* settings. The effectiveness of AHR regimens using EBDs on IAV in mucus was extremely reduced compared to that in saline, and infectious mucus containing IAV that adhered to the fingers was not effectively inactivated at 30 s. Current hand hygiene methods recommended by the CDC and WHO involve the use of disinfectants such as EBDs for 15 to 30 s (7–10). However, our results suggest that this disinfection time is insufficient for the disinfection of infectious mucus of IAV-infected patients adhered to the fingers/hands and that current contact infection prevention and AHR regimens using EBDs are not sufficient to prevent IAV outbreaks. On the other hand, AHW completely inactivated IAV in infectious mucus within 30 s, indicating that the AHW regimen is more suitable than AHR regimen for inactivation of infectious mucus, including IAV.

The liquids (culture medium, buffer solution, and saline) that are typically used as media to transfer pathogens when evaluating the effectiveness of disinfection methods have physical properties similar to those of pure water (a Newtonian fluid with extremely low viscosity). These liquids do not accurately simulate the physical properties of infectious body fluids, which include non-Newtonian fluids with relatively high viscosity, such as sputum and nasal discharge. Therefore, disinfectants (and disinfection methods) produced and approved using the currently recommended process can be insufficient for disinfection of infectious mucus, and deviations can occur between the expected effects and the actual clinical effects (7–10, 17–20). A disinfectant effectiveness assessment test under conditions reproducing the physical properties of infectious mucus should be added to the current assessment standards for disinfectant effectiveness. Next-generation disinfectants developed/approved under these new disinfectant effectiveness assessment standards will be sufficiently effective against infectious mucus and will contribute greatly to the advancement of contact infection prevention.

Since the decrease in the effectiveness of EBDs on infectious mucus is attributable to the physical properties of mucus as a hydrogel, it is expected that the difference in the effectiveness of EBDs in saline and mucus conditions will be drastically reduced if the mucus is dried completely and the gel-like characteristics are lost. In fact, IAV both in dried mucus and in dried saline was rapidly inactivated within 30 s after exposure to EBD in both *in vitro* and *in situ* inactivation tests. For infectious mucus that is completely dried on fingers, EBDs exert IAV-inactivating effects in a short time (within 30 s), as conventionally recognized. In contrast, the IAV-inactivating effects of EBDs are extremely reduced against undried gelled infectious mucus. In other words, the current AHR regimen using EBDs is ineffective against infectious mucus before the mucus dries and is insufficient as a contact infection prevention measure.

In the current ASTM/CEN disinfectant effectiveness assessment test or in previous clinical studies, the effectiveness of disinfectants is generally evaluated after a sample (infectious body fluid) is placed on the test subject’s fingers and is completely dried (17–20, 32, 33). Thus, the reduction of disinfectant effectiveness against pathogens in undried mucus has not been paid enough attention so far. In our study, the time required for mucus (sputum) samples from IAV-infected patients to completely dry was approximately 30 min in an indoor environment (temperature, 25°C; humidity, 40%). EBD cannot exert sufficient inactivating effects on infectious mucus if the mucus is not completely dry, because infectious mucus maintains a gel form. For 30 min after infectious mucus has adhered to the hand and fingers, IAV in infectious mucus cannot be inactivated by AHR using EBD and remains transmittable; this increases the risk of spreading IAV infections.

Since the WHO announced the “Five moments for hand hygiene” recommendation in 2009, medical institutions around the world have been aiming to comply with this hand hygiene requirement (11, 34). However, our study revealed that “AHR immediately after treatment for IAV infected patients” cannot sufficiently inactivate IAV in infectious mucus, and infectious IAV can remain on the hand and fingers, since the infectious mucus is not dry. If sufficient time elapses before treating the next patient, “AHR before touching the next patient” completely inactivates IAV in infectious mucus adhering to the hand and fingers, because infectious mucus is completely dried. If there is an insufficient time or no time gap before treating the next patient, IAV in infectious mucus cannot be inactivated by “AHR before touching the next patient” because infectious mucus does not have time to dry completely. In these cases, the risk of spreading IAV to the patient to be treated next will be higher than expected. In contrast, even if there is an insufficient time or no time gap before treating the next patient, “AHW before touching the next patient” can completely inactivate IAV in infectious mucus.

As described above, situations where the effectiveness of EBD in infectious mucus is reduced (i.e., the current AHR vulnerability is exposed) are limited. However, in a realistic medical setting, a sufficient time interval cannot be secured between treatments, and the next patient’s treatment is performed immediately after the current patient’s treatment in many cases. Furthermore, since there is no time margin in such cases, simple and quick AHR rather than AHW is preferred and practiced for contact infection prevention (35, 36). However, situations where the current AHR regimen is preferred and practiced are exactly the situations where AHR has minimal efficacy.

The limitations of AHR must be overcome in the future. Since nonenveloped viruses (e.g., norovirus and adenovirus) are not easily inactivated by liquid disinfectants such as EBDs, prevention of contact infection with nonenveloped viruses is difficult (37–40). Although enveloped viruses such as IAV are usually inactivated relatively easily by EBDs, those present in undried infectious mucus are not effectively inactivated. Therefore, prevention of contact infection with enveloped viruses in undried infectious mucus should be performed in the same way as with nonenveloped virus. Moreover, “Combination of means for drying mucus in a short time” and “Increase frequency of conventional AHW that washes out mucus physically instead of AHR” may also contribute to more effective contact infection prevention.

There were three limitations to this study. First, regarding the AHW regimens in this study, handwashing was practiced using running water only, without using plain or antiseptic soap. These AHW regimens were expected to have a lower pathogen inactivation effect compared to that of AHW regimens using plain or antiseptic soap. However, IAV on the fingers was completely inactivated within 30 s under all AHW conditions, showing a good IAV inactivation effect of AHW in this study. Therefore, this limitation did not affect the results and conclusions of this study. Second, the additional fluid simulation was performed with the mucus (sample no. 10) viscosity reduced to 16.7 or 12.5% of the measured value (Fig. 6; Fig. S4A). In the initial simulation of mucus conditions using the measured mucus viscosity, the time required for regions containing <31% ethanol to disappear exceeded 120 s. Times exceeding 120 s are not realistic, because the calculation time and costs are dramatically increased in the additional simulation reproducing the reaction conditions of the ethanol concentration measurement and the inactivation tests more faithfully. Therefore, the additional fluid simulation was performed with the mucus viscosity reduced. The result of the initial simulation under the same low-viscosity conditions (12.5 and 16.7% of the measured value) was as with the additional simulation (Fig. S4D); the results of actual measurement of ethanol concentrations in low-viscosity mucus (Fig. S4C) were generally compatible with the results of the additional simulation (Fig. S4A). The validity of the additional fluid simulation was confirmed, and it was demonstrated that the physical properties of mucus greatly affect the increase of ethanol concentration in mucus. Third, to estimate the time required for saline or mucus to completely dry up, the time required for samples to completely dry up on a glass plate was measured in an indoor environment. In the future, it will be necessary to establish a method for measuring the complete drying time of infectious mucus on human skin. Depending on the results of the future study, the complete drying time may vary slightly from a minimum of “30 min,” as shown in this study.

In this study, we showed that the disinfection effectiveness of EBDs against IAV decreases drastically in mucus; we further proposed a possible mechanism for this phenomenon. Additionally, we revealed the limitation of AHR, the main means of current hand hygiene, with reference to undried infectious mucus. These findings will greatly contribute not only to the development of a more effective method of preventing IAV outbreaks but also to the advancement of current hand hygiene and contact infection prevention strategies.

## MATERIALS AND METHODS

### Viruses and cells.

Madin-Darby canine kidney (MDCK) cells were purchased from the Riken BioResource Center Cell Bank (Ibaragi, Japan) and cultured in minimal essential medium (MEM; Sigma, St. Louis, MO, USA) supplemented with 10% fetal bovine serum and standard antibiotics (6, 41). IAV (PR8; A/Puerto Rico/8/1934; H1N1) was cultured in MDCK cells and stored as a working stock at −80°C. Viral titers were measured by focus-forming assays in MDCK cells and are expressed as the number of focus-forming units (FFU)/ml (21).

### Collection of sputum samples and preparation of mucus.

**FIG 7:**
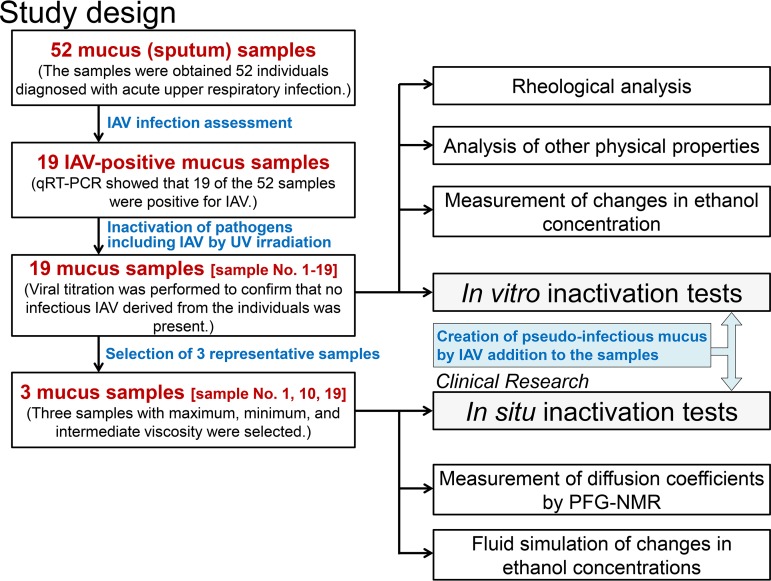
Outline of the study.

Figure 7 shows the overall workflow of this study. Mucus samples (sputum samples [≥2 g]) were obtained from 52 individuals who were diagnosed with acute upper respiratory tract infection at the Kyoto Prefectural University of Medicine Hospital and its affiliated hospitals between September 2016 and March 2018. Individuals <20 years old, with chronic respiratory illness, or who were taking expectorants were excluded.

The 52 mucus samples were evaluated by quantitative reverse transcription-PCR (qRT-PCR) to distinguish between IAV-infected and IAV-uninfected samples. Nucleic acids were extracted from the mucus samples with a PureLink RNA minikit (Thermo Fisher, Waltham, MA, USA), and cDNA was prepared using a ReverTra Ace qPCR RT master mix (Toyobo, Osaka, Japan) (6, 21). IAV was detected and quantified by qRT-PCR with a Thunderbird SYBR qPCR mix (Toyobo). IAV-specific PCR was performed with an IAV matrix protein gene-specific primer set (M30F, 5′-TTCTAACCGAGGTCGAAACG-3′; M264R2, 5′-ACAAAGCGTCTACGCTGCAG-3′) designed by the National Institute of Infectious Diseases (Tokyo, Japan). Results of the qRT-PCR analysis showed that 19 of the 52 samples were positive for IAV, and these 19 samples were used in this study.

To completely inactivate infectious IAV derived from the individuals, the samples were irradiated with UV light (10 mJ/cm^2^). Viral titration with MDCK cells was performed to confirm that no infectious IAV was present. Bubbles were removed from irradiated samples by centrifugation at low speed to avoid destroying the mucous structure.

### Rheological analysis.

The viscosity of the mucus samples was measured with a DHR-1 controlled-stress rheometer (TA Instruments, New Castle, DE, USA) (42). A cone plate geometry (20 mm, 2°) was used at 25°C. A solvent trap containing distilled water prevented sample dehydration during measurement (43). Mucus (400 mg) was loaded onto the rheometer and left for 5 min to enable relaxation to the original gel structure. Steady-flow viscosities were measured with the flow-sweep mode (steady-flow measurement). Steady-flow viscosity (in Pa s) and shear stress (in Pa) were determined with the Rheology Advantage data-analysis software (TRIOS Software, TA Instruments) for a range of shear rates (0.01, 0.016, 0.025, 0.04, 0.063, 0.1, 0.16, 0.25, 0.4, 0.63, 1.0, 1.6, 2.5, 4.0, 6.3, 10, 16, 25, 40, 63, and 100 s^−1^). Steady-flow viscosity was calculated as shear stress divided by shear rate.

### Analysis of other physical properties.

The contact angles of pure water, saline, and sputum samples on a glass surface were measured with a contact angle meter (Asumigiken, Tokyo, Japan), and surface tensions were measured with a surface tension meter (Asumigiken).

### Analysis of complete drying time of saline and mucus.

The time required for 5 μl of saline or mucus samples to completely dry and solidify on a glass plate was measured. In an indoor environment (temperature, 25°C; humidity, 40%), 5 μl of each sample was placed on a glass dish and weighed over time. The weight decreased gradually due to the drying of the sample, and the time when the weight ceased to decrease was defined as the “complete drying time.” An analytical balance (XFR-135; Shinko Denshi, Tokyo, Japan) was used to measure the weight of the samples.

### Measurement of diffusion coefficients by PFG-NMR.

Three representative mucus samples (no. 1, 10, and 19) were selected, and the ethanol diffusion coefficients in these samples and those in saline were measured by pulsed-field gradient nuclear magnetic resonance (PFG-NMR). Samples were prepared by mixing deuterium oxide at 99.9 atom% D (D_2_O; Isotech Laboratories, Inc., Champaign, IL, USA) and 99.5% ethanol (Kanto Chemical, Tokyo, Japan) into the mucus samples. Here, D_2_O minimized the signal intensity from the OH group in ^1^H NMR measurement. ^1^H NMR spectra of the mucus-alcohol-D_2_O mixtures were acquired at 298 ± 1 K. All NMR measurements were conducted using a Diff-30 diffusion probe on a Bruker Avance 600 spectrometer (Hitachi, Tokyo, Japan) operating at a ^1^H resonance frequency of 600.13 MHz. The chemical shifts were calibrated using the ^1^H resonance of tetramethylsilane as an external reference. PFG-NMR self-diffusion measurements were carried out using the pulsed-gradient spin-echo sequence, starting from the minimum value of 0.05 midpoint temperature (*T_m_*^−1^). The maximum gradient strength was adjusted experimentally to 2.0 to 3.0 *T_m_*^−1^ to maximize the accuracy of fitting from signal attenuation. The diffusion time (Δ) was fixed to 20 ms, and the gradient-pulse duration (δ) was set to 1 ms. For all samples, 5-mm NMR glass tubes were used; the tubes were sealed to prevent evaporation of the solvent. In total, eight signal averages were acquired for each gradient increment (*g*) for a total of 16 increments. The recycle time was 5 s, resulting in a total acquisition time for an individual PFG-NMR data set of <12 min.

The area (Ψ) of the respective resonance peaks was measured as a function of the gradient strength (*g*), and the self-diffusion coefficient (*D*) was determined using equation 1:

(1) $$\Psi =\text{exp}\left(-{\text{γ}}^{2}{\text{δ}}^{2}g^{2}\left[\Delta -\text{δ}/3\right]\right)$$

where γ is the gyromagnetic ratio of the ^1^H nucleus.

After measurement of the self-diffusion coefficients of the water and ethanol molecules, the interdiffusion coefficient (*D*) of the water-ethanol system was calculated from the self-diffusion coefficients of water (*D*_H2O_) and ethanol (*D*_EtOH_). *D* was determined using Darken’s equation, as follows (2, 44, 45):

(2) $$D=N_{\text{EtOH}}\times D_{{\text{H}}_{2}\text{O}}+N_{{\text{H}}_{2}\text{O}}\times D_{\text{EtOH}}$$

where *N* is the mole fraction.

### Measurement of changes in ethanol concentration with a modified reflectometer.

Changes in ethanol concentration in saline or mucus were measured after addition of 80% (wt/wt) ethanol-based disinfectant (EBD) (see Fig. S5A in the supplemental material). Ethanol concentrations were measured with a digital ethanol densitometer (ATAGO, Tokyo, Japan), which was modified for this measurement. The digital ethanol densitometer is a refractometer that measures the ethanol concentration at the center of a sample placed on a prism in a hemispherical region with a diameter of ∼1 mm; this enables measurement of changes in ethanol concentration over time in the region of the sample with the lowest ethanol concentration. For this manual procedure, 95 μl of EBD was added as slowly as possible to a 5-μl sample placed on a prism. The densitometer was then used for measurement of the ethanol concentration over time. A fitting curve was prepared from the ethanol concentration at each time point, and the time for the ethanol concentration to reach 5, 10, 15, 20, 25, 30, 31, 35, and 40% was calculated from the fitting curve. Three independent experiments were performed, and the results are expressed as the mean ± standard deviation (SD).

10.1128/mSphere.00474-19.5 FIG S5 Protocols for measurement of the effects of an ethanol-based disinfectant (EBD) on influenza A virus (IAV) in saline or mucus. (A) Changes in the ethanol concentration in the center region (the region with the lowest ethanol concentration) of saline or mucus were measured using a digital ethanol densitometer modified for this analysis. EBD was added to saline or mucus, and the ethanol concentration was measured over time. (B) *In vitro* inactivation tests for evaluation of EBD efficacy. (C and D) Clinical research (*in situ* inactivation tests) on the efficacy evaluation of antiseptic hand rubbing (AHR [C]) and antiseptic hand washing (AHW [D]). The situation in which infectious mucus discharged from IAV-infected patients adheres to the fingers of medical staff was faithfully reproduced. (E) The additional fluid simulation of changes in ethanol concentrations using OpenFOAM software. The conditions of the simulation were set to reproduce the reaction conditions of EBD and saline or mucus in the inactivation test as much as possible. Figure S6 shows the physical characteristic values used for this fluid analysis. Download FIG S5, TIF file, 2.8 MB. Copyright © 2019 Hirose et al. 2019 Hirose et al. This content is distributed under the terms of the Creative Commons Attribution 4.0 International license.

10.1128/mSphere.00474-19.6 FIG S6 Physical property values used for fluid analysis. The physical properties of saline and mucus used in the simulation of changes in ethanol concentration with the OpenFOAM software are shown. Download FIG S6, TIF file, 1.2 MB. Copyright © 2019 Hirose et al. 2019 Hirose et al. This content is distributed under the terms of the Creative Commons Attribution 4.0 International license.

### *In vitro* inactivation tests for evaluation of EBD efficacy against IAV.

Tests for inactivation of IAV were carried out according to the following protocols (Fig. S5B), with EBD containing 80% (wt/wt) ethanol; established guidelines were followed for these tests (EN14476:2013/FprA1:2015) (19, 38, 46–48). IAV was mixed with saline or mucus prior to EBD exposure. Briefly, 95 μl of EBD or PBS was added to 5 μl of a mixture of IAV and mucus or saline (final viral titer, 1.0 × 10^6^ FFU/ml) in 24-well plates and incubated at 25°C for 15, 30, 60, 120, 180, 210, 240, or 300 s prior to neutralization by dilution with 900 μl MEM; titration of IAV was then performed. The survival ratio was calculated as the ratio of the titer after incubation with disinfectant to that measured after incubation with PBS; this ratio indicated the proportion of IAV that was not inactivated by the disinfectant. A fitting curve was prepared using the survival ratio at each time point, and complete inactivation time (time required for IAV to be completely inactivated) was calculated from the fitting curve. Complete inactivation was defined as a survival ratio below 10^−5^. In addition, the mixture of IAV and saline or mucus was dried completely by letting the mixture stand for 40 min before EBD exposure, and the same inactivation test described above was carried out. For each measurement, three independent experiments were performed, and the results are expressed as the mean ± standard deviation.

### Clinical research (*in situ* inactivation tests for evaluation of EBD efficacy against IAV).

Clinical research was performed with 10 test subjects who provided informed consent. Three representative mucus samples were selected (no. 1, 10, and 19), and the effectiveness of EBD against IAV in mucus or saline was examined using *in situ* inactivation tests conducted under established guidelines (finger pad protocol, ASTM E1838; hand wash protocol, EN14476) (32, 33, 47).

First, clinical research concerning the efficacy evaluation of AHR was performed (Fig. S5C). IAV was mixed with saline or mucus (final viral titer, 1.0 × 10^6^ FFU/ml) and applied in 5-μl aliquots to washed and disinfected fingertips of test subjects in a marked area. A tube containing 95 μl of EBD was placed on the contaminated area and incubated for 30, 60, 120, or 240 s prior to neutralization and elution with 500 μl MEM; IAV was then titrated. The mixture of IAV and saline or mucus on fingertips was completely dried by standing for 40 min before exposure to EBD, and the same efficacy evaluation described above was carried out.

Next, clinical research concerning the efficacy evaluation of AHW was performed (Fig. S5D). IAV was mixed with saline or mucus (final viral titer, 1.0 × 10^6^ FFU/ml) and applied in 5-μl aliquots to washed and disinfected fingertips of test subjects in a marked area. The contaminated fingers were hand washed with running water for 30, 60, 120, or 240 s prior to neutralization and elution with 500 μl MEM; IAV was then titrated. The mixture of IAV and saline or mucus on fingertips was completely dried by standing for 40 min before hand washing, and the same efficacy evaluation described above was carried out.

### OpenFOAM simulation of changes in ethanol concentrations.

OpenFOAM is an open-source software for continuum physics simulations and computational fluid dynamics. The InterMixingFoam solver of OpenFOAM 2.2.2 was used for this analysis. InterMixingFoam solved the incompressible Navier-Stokes equation and solved the conservation law of the volume fraction of each fluid (gas and two types of liquid). The volume fractions (α_1_, α_2_, and α_3_) satisfied equation 3:

(3) $${\text{α}}_{1}+{\text{α}}_{2}+{\text{α}}_{3}=1$$

In this analysis, the volume fraction, α_1_, was air, α_2_ was an 80% ethanol aqueous solution, and α_3_ was saline or mucus. The density (ρ) and viscosity (μ) of the whole fluid could be obtained from equation 4 and equation 5 using the density, viscosity, and volume fraction of each fluid.

(4) $$\text{ρ}={\text{ρ}}_{1}{\text{α}}_{1}+{\text{ρ}}_{2}{\text{α}}_{2}+{\text{ρ}}_{3}{\text{α}}_{3}$$

(5) $$\text{μ}={\text{μ}}_{1}{\text{α}}_{1}+{\text{μ}}_{2}{\text{α}}_{2}+{\text{μ}}_{3}{\text{α}}_{3}$$

Additionally, the ethanol concentration (χ) could be obtained from equation 6:

(6) $$\text{χ}=\left(80\cdot {\text{ρ}}_{2}{\text{α}}_{2}\right)/\left({\text{ρ}}_{1}{\text{α}}_{1}+{\text{ρ}}_{2}{\text{α}}_{2}+{\text{ρ}}_{3}{\text{α}}_{3}\right)$$

The entire fluid domain was analyzed using the Navier-Stokes equations, which include the law of conservation of mass (equation 7) and the law of conservation of momentum (equation 8).

(7) $$\frac{\partial \text{ρ}}{\partial t}+\frac{\partial }{\partial x_{i}}(\text{ρ}U_{i})=0$$

(8) $$\frac{\partial \text{ρ}U_{i}}{\partial t}+\frac{\partial }{\partial x_{j}}(\text{ρ}U_{i}U_{j})=-\frac{\partial \text{ρ}}{\partial x_{i}}+\text{μ}\frac{\partial }{\partial x_{i}}\frac{\partial U_{i}}{\partial x_{j}}+\text{ρ}g_{i}+(f_{12})i+(f_{13})i$$

where *g_i_* is the gravitational acceleration and *f*_12_ and *f*_13_ are the surface tensions.

The surface tension (*f*_12_) between the gas α_1_ and the liquid α_2_ and the surface tension (*f*_13_) between the gas α_1_ and the liquid α_3_ can be obtained by the following equations, respectively (equation 9 and equation 10):

(9) $$f_{12}={\text{σ}}_{12}(\nabla \cdot {\overset{\to }{n}}_{12}){\overset{\to }{n}}_{12}$$

(10) $$f_{13}={\text{σ}}_{13}(\nabla \cdot {\overset{\to }{n}}_{13}){\overset{\to }{n}}_{13}$$

where *n*_12_ and *n*_13_ are normal vectors of the interface between the α_1_ and α_2_ and α_1_ and α_3_, respectively, and σ_12_ and σ_13_ are the surface tension coefficients of the interface between α_1_ and α_2_ and gas α_1_ and α_3_, respectively.

Finally, α_1_ and α_2_ were obtained by equation 11 and equation 12:

(11) $$\frac{{\text{α}}_{2}}{d1}+U_{i}\frac{\partial {\text{α}}_{1}}{\partial x_{i}}=0$$

(12) $$\frac{{\text{α}}_{2}}{\mathrm{dt}}+U_{i}\frac{\partial {\text{α}}_{2}}{\partial x_{i}}+D\frac{\partial^{2}{\text{α}}_{2}}{\partial x_{i}^{2}}=0$$

where *D* is an interdiffusion coefficient. α_3_ was obtained from equation 3.

In the OpenFOAM code, the value of *D* was set to zero in places where α_2_ and α_3_ did not exist.

First, the initial simulation reproducing a mixing of 20 μl of saline or mucus and 480 μl of EBD in a 96-well plate was conducted (Fig. 3A). Fluid simulation was carried out for a total of four conditions using the physical property values of saline or those of the three mucus samples (no. 1, 10, and 19).

Next, the conditions of the additional simulation were set to reproduce the reaction conditions of EBD and saline or mucus in the inactivation tests as much as possible. Briefly, cubic meshes with sides of 0.2 mm were used for the simulation, and 95 μl of 80% ethanol was added at a flow rate of 250 μl/s from a height of 6.0 mm above 5 μl of saline or mucus (Fig. S5E). The physical properties of the sample (no. 10) with a median viscosity among all 19 mucus samples were adopted as the physical properties of the mucus used in the simulation. In the initial simple simulation of mucus conditions using the measured viscosity of mucus, the time required for regions containing <31% ethanol to disappear exceeded 120 s. Times exceeding 120 s are not realistic because the calculation time and cost are dramatically increased. Therefore, fluid simulation was performed with the mucus (sample no. 10) viscosity reduced to 16.7 or 12.5% of the measured value.

The physical properties and ethanol diffusion coefficient of the saline and mucus used in all simulations are shown in Fig. S6 in the supplemental material.

### Ethical considerations.

Informed consent was obtained from all participants. The study protocol was reviewed and approved by the Institutional Review Board of the Kyoto Prefectural University of Medicine (ERB-C-716-2, UMIN000030152).

### Statistical analysis.

Data were analyzed using GraphPad Prism 7 software (GraphPad, La Jolla, CA, USA). Differences between the means of continuous variables were evaluated by Student's *t* test. Pearson’s correlation coefficient was used to assess the correlation between viscosity and 10, 20, and 31% ethanol concentration achievement time or between complete inactivation time and 31% ethanol concentration achievement time. For clinical data, the Wilcoxon signed rank test was performed. In this clinical study, under the condition that the Wilcoxon signed-rank test was performed under a normal distribution with an expected difference between log reductions of 2 and a standard deviation of 1, the required number of subjects was calculated to be 10 by the Monte Carlo simulation. All reported *P* values are two sided; *P* values of <0.05 were considered significant.
