# Supplementary material for: Situations Leading to Reduced Effectiveness of Current Hand Hygiene against Infectious Mucus from Influenza Virus-Infected Patients
Source: mSphere. 2019 Sep 18;4(5):e00474-19. doi: 10.1128/mSphere.00474-19 (PMC6751490; doi:10.1128/mSphere.00474-19)
Supplement: TABLE S1 [file mSphere.00474-19-st001.docx]

**Table S1. Viscosity (Pa·s) of mucus (19 mucus samples).**

| **Shear rate (s^-1^)** | **1** | **2** | **3** | **4** | **5** | **6** | **7** | **8** | **9** | **10** | **11** | **12** | **13** | **14** | **15** | **16** | **17** | **18** | **19** |
| --- | --- | --- | --- | --- | --- | --- | --- | --- | --- | --- | --- | --- | --- | --- | --- | --- | --- | --- | --- |
| **0.01** | 54.203 | 64.704 | 3.8651 | 10.915 | 30.524 | 17.832 | 157.66 | 47.097 | 13.134 | 18.269 | 17.432 | 17.232 | 19.946 | 22.393 | 30.784 | 19.966 | 3.5246 | 9.2047 | 9.0574 |
| **0.0159557** | 49.282 | 51.622 | 4.0813 | 10.175 | 23.804 | 16.641 | 153.63 | 50.756 | 12.757 | 18.306 | 14.638 | 16.23 | 18.288 | 19.219 | 25.366 | 17.072 | 5.0009 | 8.4721 | 8.5831 |
| **0.025176** | 44.135 | 37.039 | 4.0344 | 9.4149 | 17.256 | 12.889 | 142.84 | 50.642 | 11.357 | 15.633 | 11.805 | 14.07 | 15.539 | 15.284 | 19.429 | 13.508 | 5.7328 | 7.3669 | 7.7921 |
| **0.039838** | 38.426 | 24.452 | 3.6309 | 8.4544 | 12.244 | 9.2542 | 118.65 | 46.318 | 8.8366 | 11.92 | 8.9821 | 11.395 | 12.165 | 10.996 | 13.696 | 9.8118 | 5.277 | 5.9235 | 6.6615 |
| **0.0631919** | 31.95 | 15.751 | 3.2753 | 7.2397 | 8.5939 | 6.5411 | 87.499 | 38.118 | 6.2161 | 8.5216 | 6.5552 | 8.7413 | 8.901 | 7.4885 | 8.9687 | 6.7759 | 4.5084 | 4.3869 | 5.3216 |
| **0.1** | 25.274 | 10.354 | 3.0196 | 5.9685 | 5.9185 | 4.6397 | 62.278 | 29.321 | 4.1773 | 5.9799 | 4.6781 | 6.3765 | 6.1515 | 5.0197 | 5.647 | 4.5881 | 3.4678 | 2.9782 | 4.0202 |
| **0.159557** | 19.044 | 6.8077 | 2.5974 | 4.7032 | 3.945 | 3.1456 | 43.088 | 22.118 | 2.6928 | 4.1598 | 3.2388 | 4.5431 | 4.1404 | 3.2634 | 3.4902 | 2.9656 | 2.4119 | 2.0115 | 2.825 |
| **0.25176** | 13.508 | 4.6118 | 2.1653 | 3.5656 | 2.6863 | 2.0165 | 28.78 | 16.235 | 1.7454 | 2.7852 | 2.3841 | 3.1657 | 2.7289 | 2.1994 | 2.1563 | 2.0211 | 1.7128 | 1.3842 | 1.9794 |
| **0.39838** | 9.3244 | 3.2419 | 1.67 | 2.522 | 1.6449 | 1.3163 | 19.649 | 11.166 | 1.4129 | 1.7062 | 1.6968 | 2.0677 | 1.7179 | 1.5425 | 1.48 | 1.4173 | 1.1898 | 0.9416 | 1.3594 |
| **0.631919** | 6.3986 | 2.3047 | 1.176 | 1.7677 | 1.2637 | 1.0454 | 13.739 | 7.4592 | 0.9556 | 1.1902 | 0.9144 | 1.2297 | 1.0174 | 1.1512 | 1.1239 | 1.0664 | 0.9269 | 0.7559 | 0.7475 |
| **1** | 4.6127 | 1.491 | 0.8924 | 1.3005 | 0.8171 | 0.9578 | 9.3371 | 5.1736 | 1.1595 | 0.9204 | 0.683 | 0.8935 | 0.8043 | 0.834 | 0.703 | 0.7753 | 0.6619 | 0.5016 | 0.4497 |
| **1.59557** | 3.5661 | 1.1239 | 0.654 | 0.994 | 0.6038 | 0.6668 | 6.4214 | 3.7799 | 0.8246 | 0.5991 | 0.4684 | 0.7503 | 0.4884 | 0.5016 | 0.4577 | 0.4577 | 0.3744 | 0.2847 | 0.4415 |
| **2.5176** | 2.6229 | 0.8728 | 0.4765 | 0.8156 | 0.5773 | 0.5096 | 4.3703 | 2.5802 | 0.4249 | 0.3969 | 0.494 | 0.6075 | 0.5418 | 0.3253 | 0.4361 | 0.2922 | 0.2352 | 0.2395 | 0.3411 |
| **3.9838** | 1.9133 | 0.6725 | 0.4137 | 0.7084 | 0.4435 | 0.3697 | 2.9529 | 1.6777 | 0.2917 | 0.3782 | 0.3123 | 0.3567 | 0.2694 | 0.2749 | 0.2273 | 0.2505 | 0.1972 | 0.1643 | 0.1505 |
| **6.31919** | 1.2733 | 0.5104 | 0.3783 | 0.549 | 0.3754 | 0.2832 | 1.9351 | 1.1674 | 0.2674 | 0.2335 | 0.2537 | 0.2786 | 0.225 | 0.1741 | 0.1815 | 0.1554 | 0.1125 | 0.0886 | 0.1172 |
| **10** | 0.7945 | 0.4279 | 0.3057 | 0.406 | 0.2671 | 0.2303 | 1.289 | 0.7836 | 0.1896 | 0.1816 | 0.1728 | 0.1934 | 0.1529 | 0.1165 | 0.1201 | 0.1024 | 0.0732 | 0.0559 | 0.0726 |
| **15.8732** | 0.4905 | 0.371 | 0.2289 | 0.3143 | 0.1878 | 0.1942 | 0.8484 | 0.5283 | 0.1328 | 0.1275 | 0.1256 | 0.1236 | 0.1059 | 0.0836 | 0.0803 | 0.0731 | 0.054 | 0.0406 | 0.0441 |
| **25.1313** | 0.4261 | 0.2614 | 0.2059 | 0.2332 | 0.1497 | 0.1494 | 0.5632 | 0.3596 | 0.0978 | 0.0958 | 0.09 | 0.0867 | 0.0761 | 0.0596 | 0.0577 | 0.0512 | 0.0357 | 0.0293 | 0.0321 |
| **39.8225** | 0.3596 | 0.1983 | 0.1579 | 0.1508 | 0.1271 | 0.1133 | 0.3786 | 0.2457 | 0.0752 | 0.0738 | 0.0667 | 0.0635 | 0.0564 | 0.0433 | 0.0427 | 0.0365 | 0.0233 | 0.0207 | 0.0236 |
| **63.1205** | 0.2621 | 0.1904 | 0.1289 | 0.1099 | 0.0965 | 0.0858 | 0.2574 | 0.1701 | 0.0565 | 0.0582 | 0.0483 | 0.0489 | 0.0401 | 0.0323 | 0.0313 | 0.0261 | 0.017 | 0.0149 | 0.0175 |
| **100** | 0.2333 | 0.1172 | 0.0721 | 0.0891 | 0.0957 | 0.0778 | 0.1764 | 0.1189 | 0.0475 | 0.042 | 0.0349 | 0.0378 | 0.0306 | 0.0245 | 0.0235 | 0.0195 | 0.0124 | 0.0117 | 0.0111 |
