# Supplementary figures and images for: Situations Leading to Reduced Effectiveness of Current Hand Hygiene against Infectious Mucus from Influenza Virus-Infected Patients
Source: mSphere. 2019 Sep 18;4(5):e00474-19. doi: 10.1128/mSphere.00474-19 (PMC6751490; doi:10.1128/mSphere.00474-19)

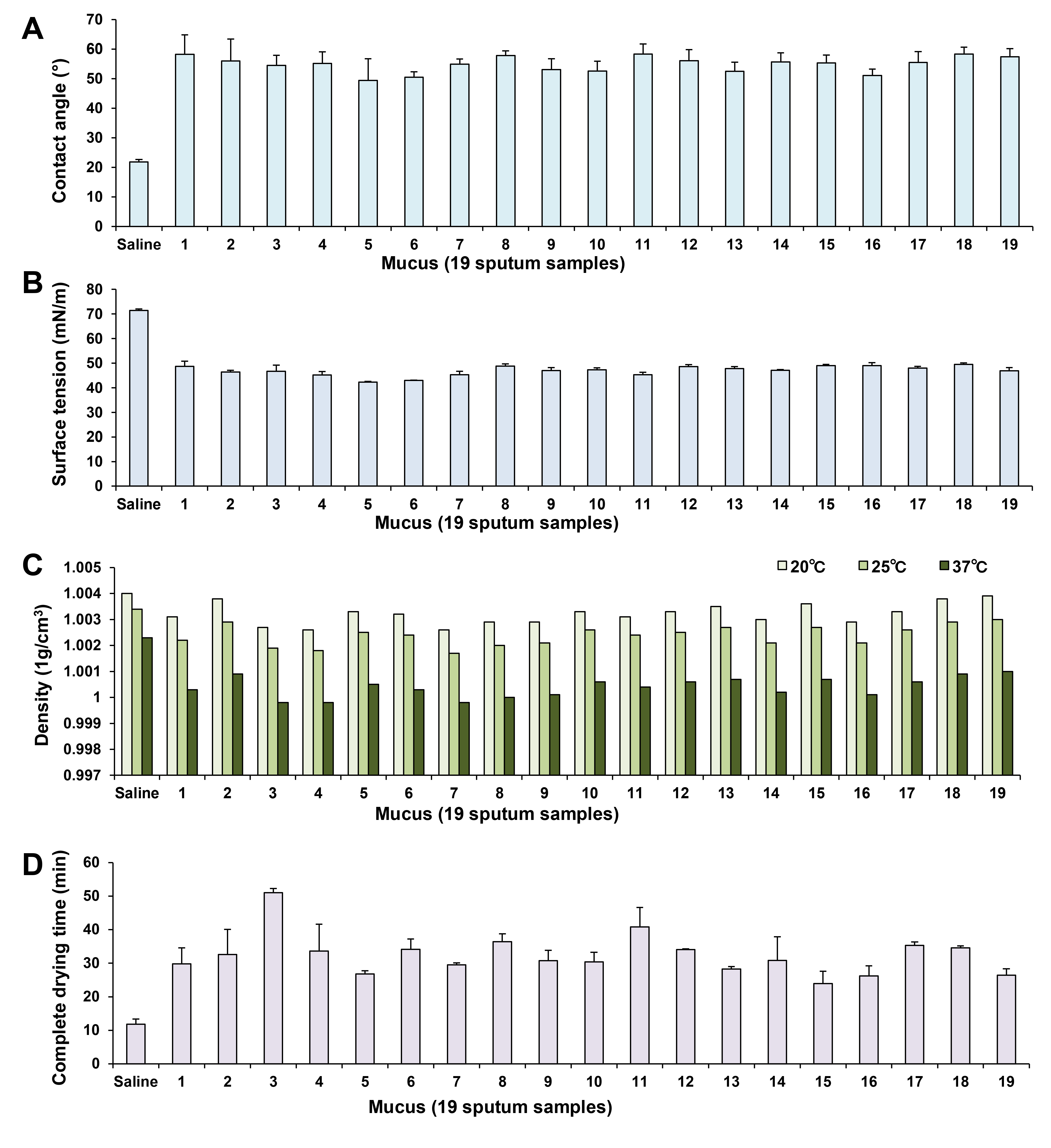

Supplement: FIG S1 [file mSphere.00474-19-sf001.tif]

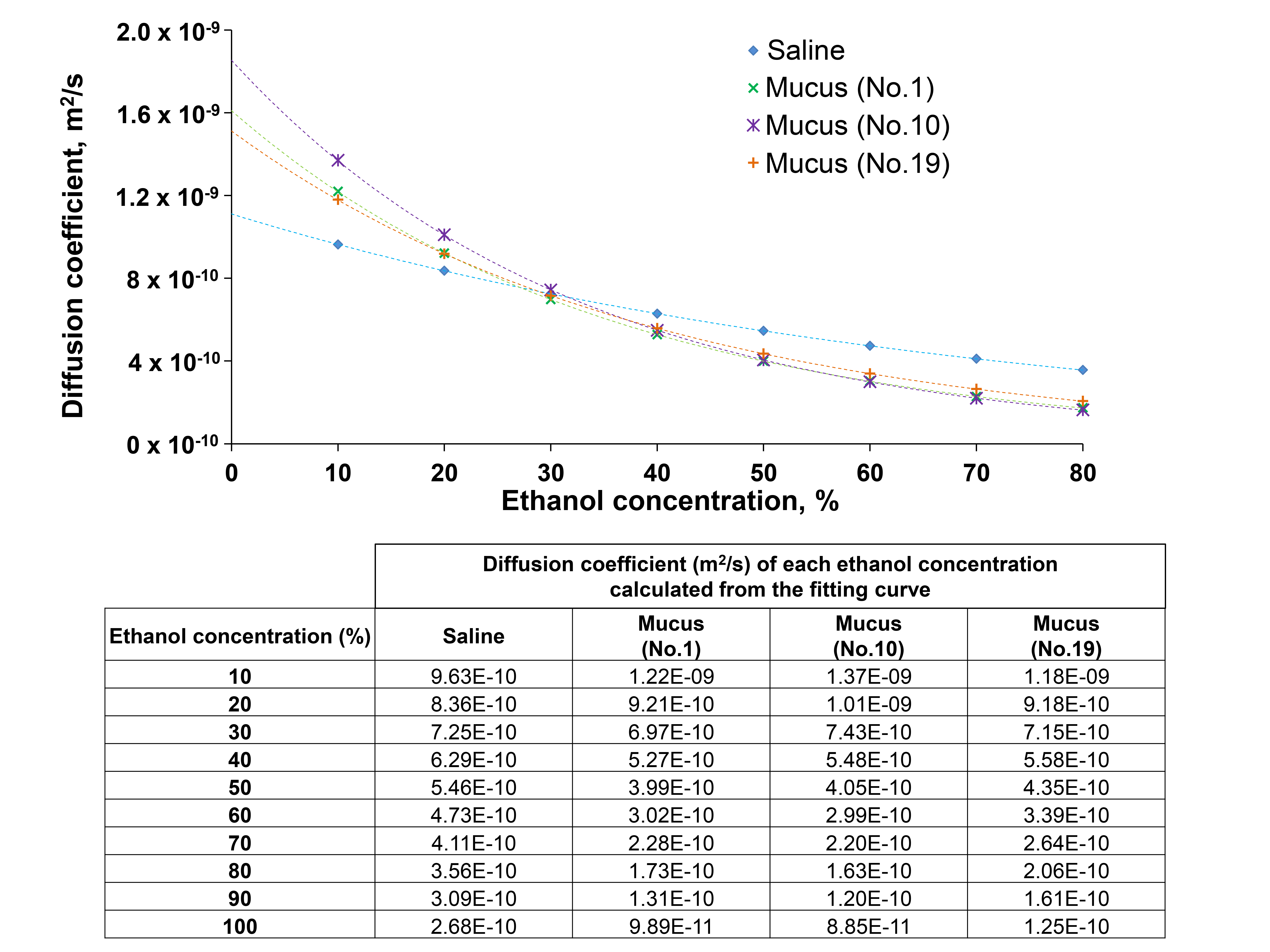

Supplement: FIG S2 [file mSphere.00474-19-sf002.tif]

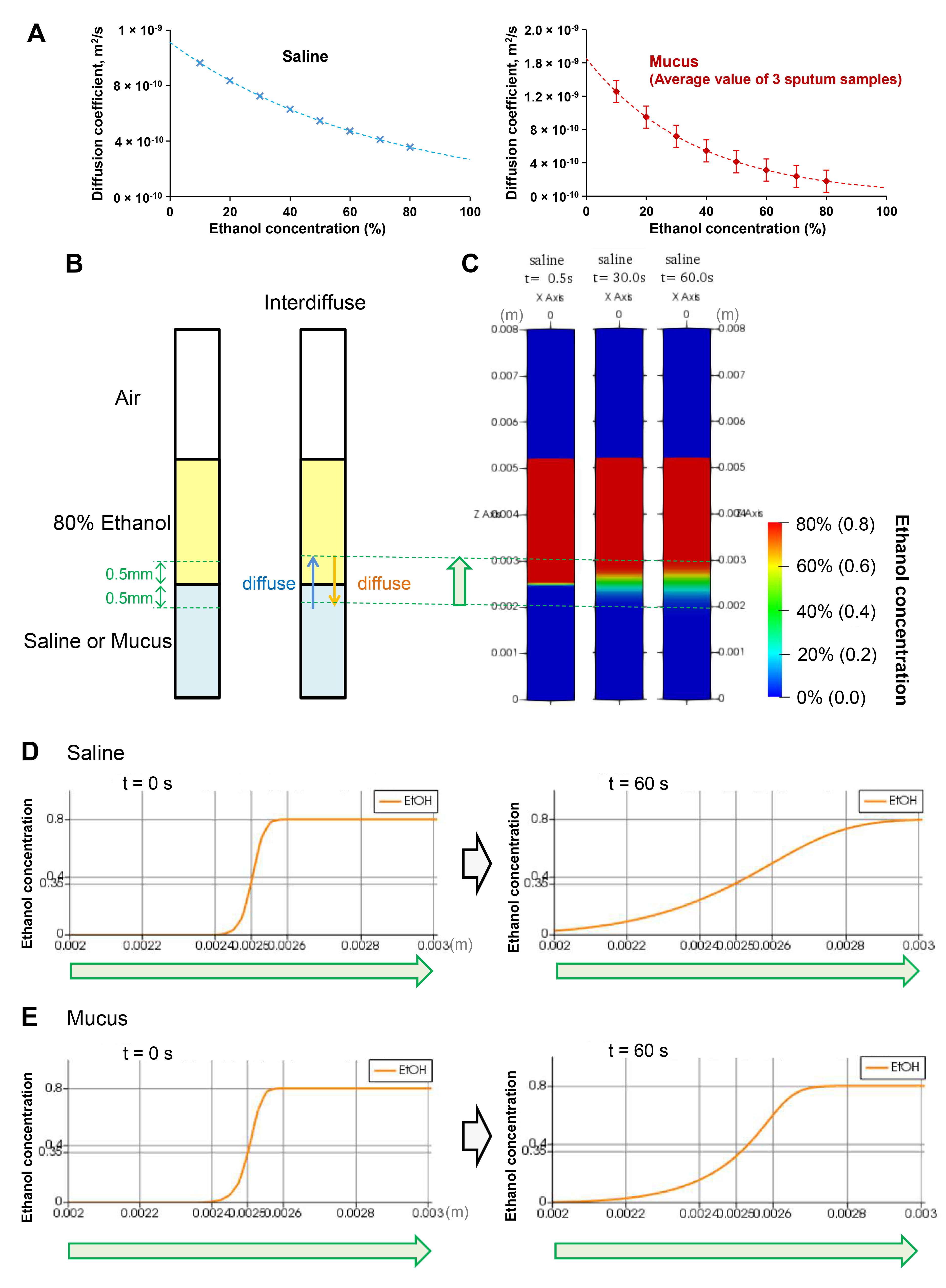

Supplement: FIG S3 [file mSphere.00474-19-sf003.tif]

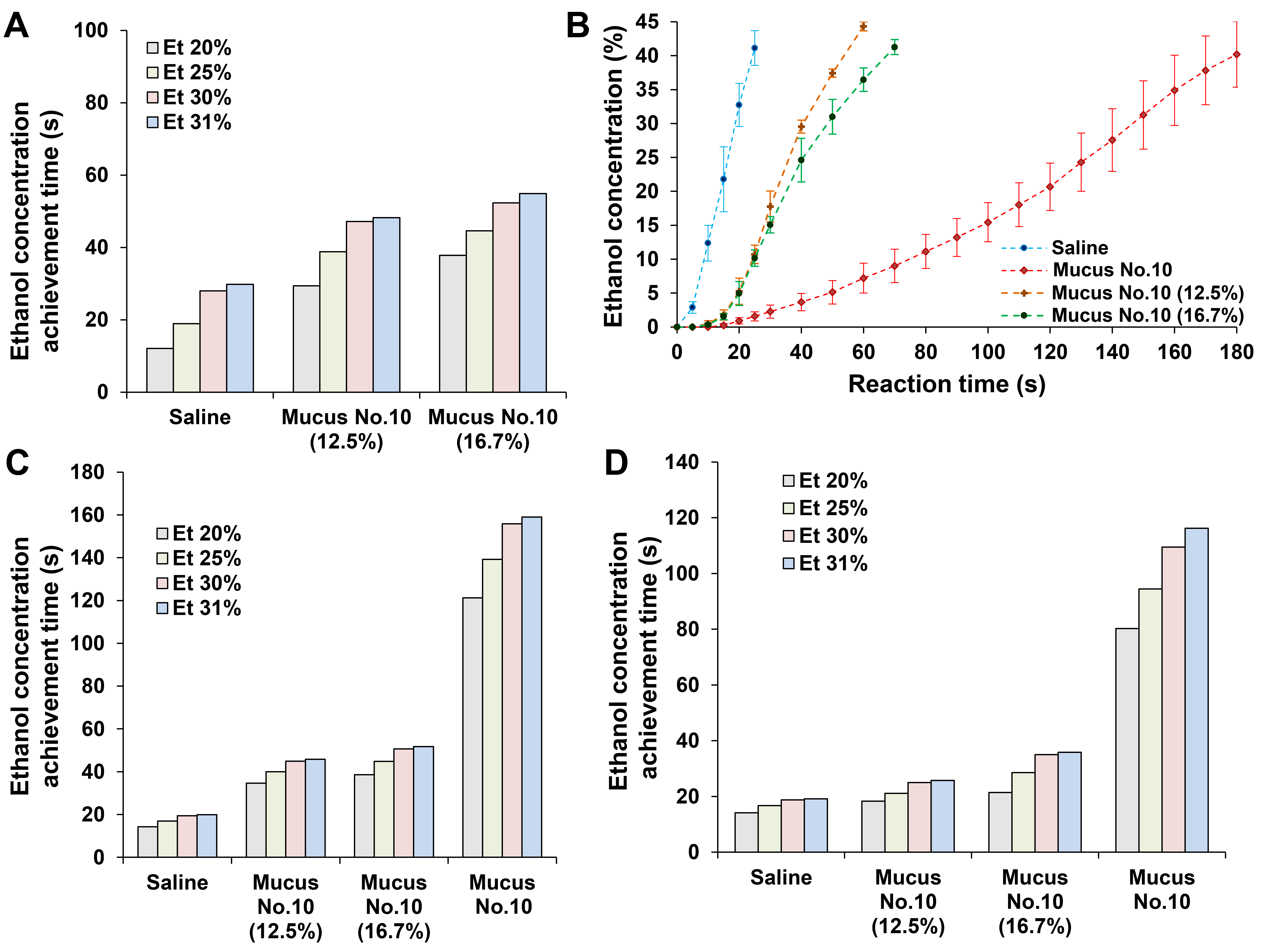

Supplement: FIG S4 [file mSphere.00474-19-sf004.tif]

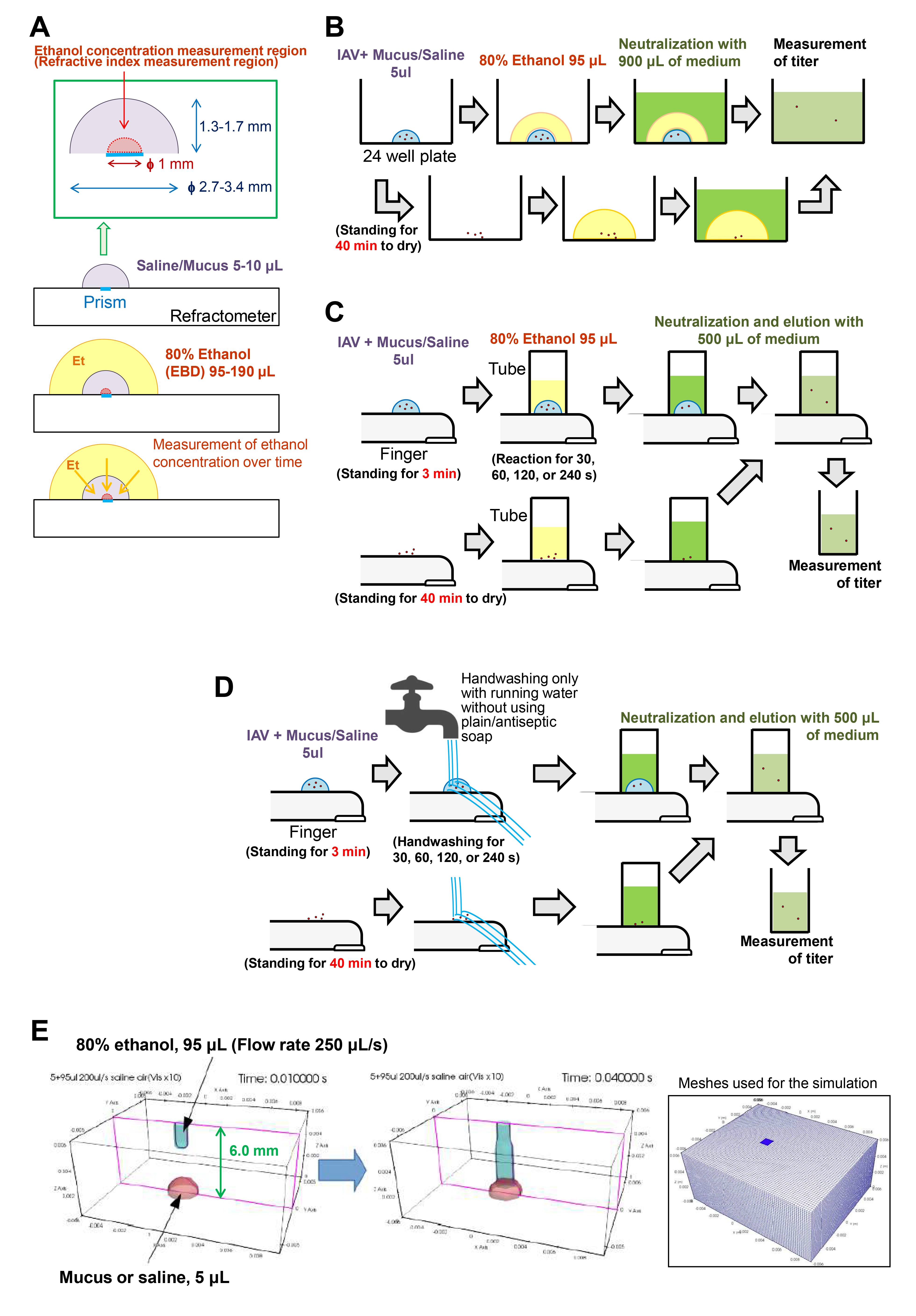

Supplement: FIG S5 [file mSphere.00474-19-sf005.tif]

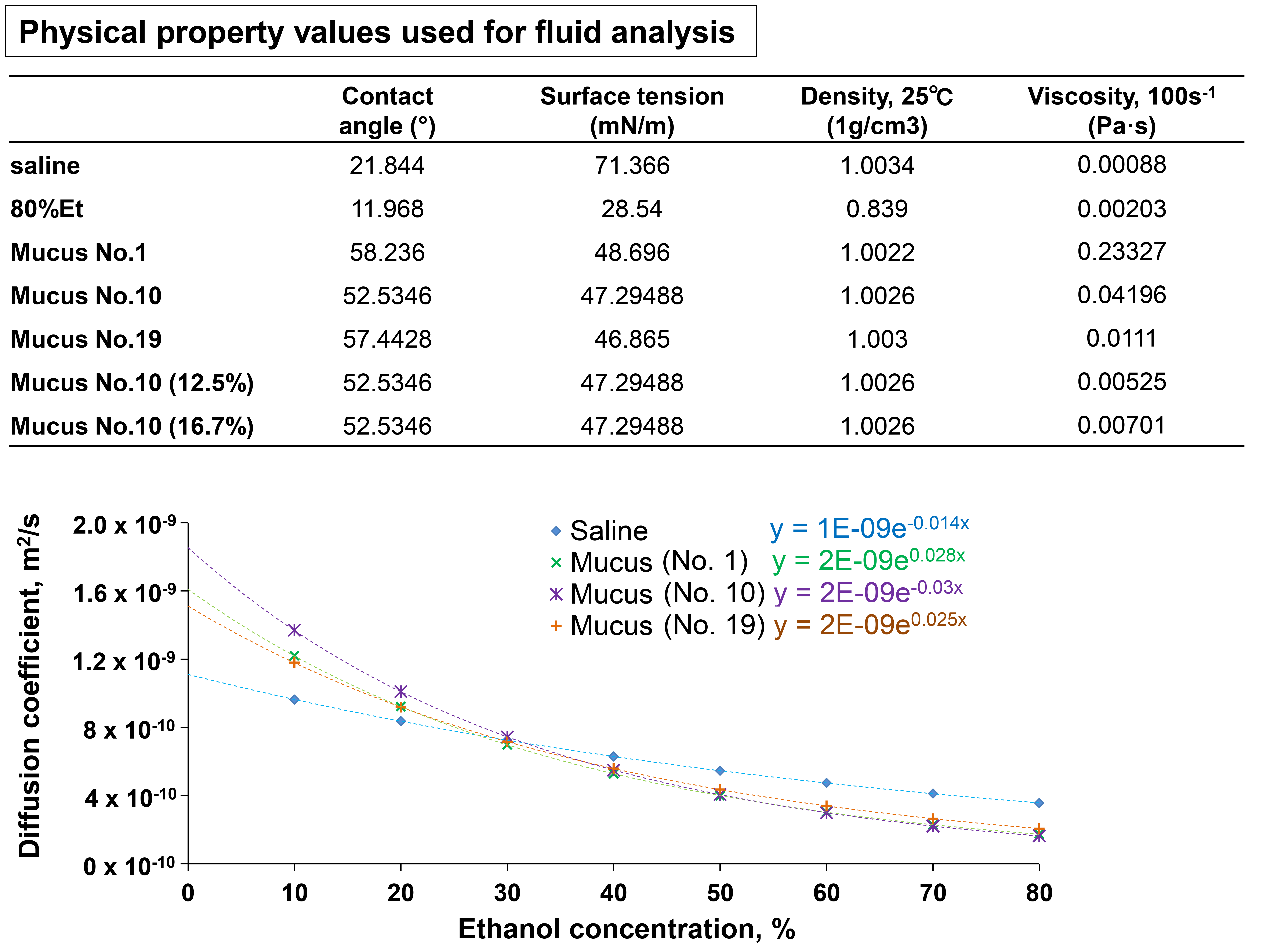

Supplement: FIG S6 [file mSphere.00474-19-sf006.tif]
